# Imaging Biomarkers of Tumour Proliferation and Invasion for Personalised Lung Cancer Therapy

**DOI:** 10.3390/jpm10040222

**Published:** 2020-11-12

**Authors:** Loredana G. Marcu

**Affiliations:** 1 Faculty of Informatics and Science, University of Oradea, 410087 Oradea, Romania; loredana@marcunet.com; 2 Cancer Research Institute, University of South Australia, Adelaide, SA 5001, Australia

**Keywords:** biomarkers, molecular imaging, non-small cell lung cancer, proliferation, cancer stem cells, circulating tumour cells, personalised treatment

## Abstract

Personalised treatment in oncology has seen great developments over the last decade, due to both technological advances and more in-depth knowledge of radiobiological processes occurring in tumours. Lung cancer therapy is no exception, as new molecular targets have been identified to further increase treatment specificity and sensitivity. Yet, tumour resistance to treatment is still one of the main reasons for treatment failure. This is due to a number of factors, among which tumour proliferation, the presence of cancer stem cells and the metastatic potential of the primary tumour are key features that require better controlling to further improve cancer management in general, and lung cancer treatment in particular. Imaging biomarkers play a key role in the identification of biological particularities within tumours and therefore are an important component of treatment personalisation in radiotherapy. Imaging techniques such as PET, SPECT, MRI that employ tumour-specific biomarkers already play a critical role in patient stratification towards individualized treatment. The aim of the current paper is to describe the radiobiological challenges of lung cancer treatment in relation to the latest imaging biomarkers that can aid in the identification of hostile cellular features for further treatment adaptation and tailoring to the individual patient’s needs.

## 1. Introduction

According to the latest Global Cancer Statistics, lung cancer is the most commonly diagnosed cancer worldwide, in both males and females (11.6% of the total cases) and the leading cause of cancer death (18.4%) [1]. Non-small cell lung cancers (as opposed to small cell lung cancers) account for about 85% of lung cancer cases and encompass adenocarcinomas, squamous cell carcinomas and large-cell undifferentiated carcinomas. Conventional therapies (surgery, chemo-radiotherapy) are being improved with new drugs and targeted agents.

While the latest technological and pharmaceutical developments have increased the therapeutic index in lung cancer, research over the last decade reveals an imperative need to include radiobiological characteristics of cellular and subcellular structures as well as the tumour microenvironment into the big picture of personalised medicine [2]. Hypoxia, proliferation, intrinsic radioresistance, and the presence of cancer stem cells are only a few, but probably the most critical features that require better management to further improve cancer treatment outcomes in general, and lung cancer treatment in particular. However, the primary tumour is not the only entity to confront. Cancer invasion and metastasis poses a therapeutic challenge by broadening the curative needs from local to systemic disease management. In this context, the identification and quantification of circulating tumour cells represent an important undertaking.

Although most of aforementioned tumour characteristics and their impact on tumour control are well known, there is still no clear-cut solution to manage treatment resistance due to high proliferative potential, the presence of cancer stem cells or circulating tumour cells that are indicative of tumour aggressiveness.

In order to tackle the above challenges, one should first identify the hostile features and then target them with the best currently available techniques. In this respect, biomarkers play a key role, as their specific design allows the identification of tumour areas that are prone to treatment resistance, thus leading the way towards personalised, targeted therapies.

The current paper focuses on the (radio) biological challenges described above applied to non-small cell lung cancer (NSCLC) and the latest imaging biomarkers that can aid in their identification, targeting and treatment outcome prediction. The main features discussed in the paper are related to tumour kinetics, via tumour proliferation and the presence of cancer stem cells, and tumour dynamics, via progression, invasion and distant metastasis through circulating tumour cells.

## 2. Tumour Proliferation and Imaging Biomarkers

### 2.1. Tumour Proliferation

Cellular proliferation is a prerequisite for tissue growth and development. Uncontrolled proliferation is characteristic of cancer cells and represents one of the hallmarks of neoplastic growth. The rate of tumour proliferation differentiates slowly proliferating from rapidly proliferating tumours, a feature that dictates the type of treatment required for tumour control. The evaluation of a tumour’s proliferative ability and of its growth kinetics are therefore critical aspects of cancer management.

Cell proliferation rate is commonly assessed through the presence in the cell nucleus of the Ki-67 monoclonal antibody, during the active phases of the cell cycle. The Ki-67 antibody labels nuclei of proliferating cells, enabling the quantification of the proliferating cell fraction within a tumour [3]. Clinical research over the years has proved Ki-67 proliferation index (or labeling index) to be a biomarker with important prognostic and predictive value in a number of cancers, including lung. The retrospective analysis of three NSCLC cohorts involving about 1500 patients showed that Ki-67 proliferation index is a highly significant and independent predictor of survival in these cancers [4]. An important aspect of the study was the individual assessment of Ki-67 correlation with each histological type of NSCLC. In this respect, the high proliferation index (PI) in adenocarcinomas was significantly associated with a worse prognosis for disease-free survival, whereas in squamous cell carcinomas the high PI was associated with better overall survival rates (cut-off value for PI of 50%). Treatment outcome among adenocarcinoma patients was further influenced by the administration of adjuvant chemo-radiotherapy, showing that patients with high PI may benefit to a higher extent from adjuvant treatment than those with low PI (cut-off value for PI of 25%).

This study showed the importance of data analysis based on histological characteristics (rather than NSCLC as a group) and the definition/validation of a Ki-67 cut-off value for each histological type of NSCLC. Furthermore, it was suggested that the predictive power of Ki-67 labeling could be enhanced by the concurrent employment of other clinical/pathological parameters as well as imaging biomarkers, which would eventually lead to better patient stratification and treatment optimisation.

Another important factor that controls cellular proliferation in lung cancers (and not only) is the epidermal growth factor receptor (EGFR). The EGFR is a transmembrane glycoprotein receptor of the ErbB family of cell surface tyrosine kinases with a role in regulating cell proliferation and apoptosis through signal transduction pathways [5]. Mutations and truncations of its extracellular matrix leads to upregulation of EGFR in several cancers, including NSCLC. Malignant as well as premalignant lesions can overexpress EGFR, with 40–80% of NSCLC patients being identified with abnormal expressions of EGFR (increased gene copy number per cell), with the highest rates seen in squamous cell carcinomas [6,7]. EGFR expression was found to be a poor prognostic factor in NSCLC, requiring efficient anti-EGFR therapies [6]. To date, EGFR-targeted therapies based on tyrosine kinase inhibitors (gefitinib, erlotinib) and monoclonal antibodies (cetuximab) have been developed with limited success, due to acquired or inherent resistance to EGFR inhibition [8]. Next to EGFR, ALK (anaplastic lymphoma kinase) translocations are known to be oncogenic drivers in NSCLC [9]. ALK translocation is associated with high sensitivity to ALK inhibitors such as crizotinib, ceritinib and alectinib [10]. Moreover, the set of mutations in these cancers is much wider. Regarding targeting avenues, ROS1 translocation is associated with a positive response to crizotinib therapy, while for BRAF mutations the combined administration of dabrafenib and trametinib, as well as the low molecular weight tyrosine kinase inhibitors vemurafenib and dabrafenib, were shown to be effective. MET mutations in lung cancer are considered to be predictors of susceptibility to the MET inhibitor crizotinib, whereas RET translocations are correlated with a positive response to targeted therapy with RET inhibitors such as cabozatinib, vandetinib, and alectinib [10]. All these mutations are important therapeutic targets, which can be identified not only in biopsy samples (given that 30% of tumour biopsies yield inadequate tissue for molecular subtyping) but also in cell-free circulating tumour DNA [11].

More recently, research into tumour proliferation has been linked to microRNAs, owing to their role in multiple biological processes, including gene regulation [12]. MicroRNAs (miRNA) are short noncoding RNAs consisting of 21–25 nucleotides that can inhibit translation of messenger RNA (mRNA) and promote mRNA degradation, thus functioning as endogenous negative gene regulators. Through posttranscriptional regulation of gene expression, miRNAs have a great impact on a number of oncogenic pathways. Recent studies demonstrated a relationship between the EGFR signaling pathway and miRNAs, showing a direct regulatory effect on EGFR [13]. Studies in NSCLC revealed the potential of miRNAs to serve in patient stratification (by risk and histology) while also predicting prognosis in early-stage NSCLC [14].

### 2.2. Imaging Biomarkers for Proliferation

Cellular kinetic parameters are important indicators of tumour proliferation before, during and after therapy, thus their quantification warrants special consideration. As shown above, the most studied proliferation markers and, consequently, the most targeted molecules related to cellular proliferation in lung cancer imaging are EGFR and Ki-67. In this regard, numerous tracers have been developed and trialed with various results [15].

#### 2.2.1. Positron Emission Tomography (PET) Imaging Biomarkers

Fluorodeoxyglucose-F18 (18F-FDG) is the most commonly used PET imaging radiotracer, being an indicator of tumour activity via glucose metabolism, and has an established role in tumour staging and treatment response monitoring. Its role in the assessment of tumour proliferation was also researched, with a considerable number of studies examining the potential of 18F-FDG in predicting EGFR mutation status in NSCLC patients. In a retrospective clinical study involving 109 NSCLC patients, Chen et al. showed that EGFR mutation decreases cellular accumulation of FDG via the NOX4/ROS/GLUT1 axis [16]. The SUVmax values in the cohort with EGFR mutations were significantly lower (6.52 mean value) than in the wild-type EGFR cohort (9.37 mean value, *p* < 0.001). Similarly, in a study of 102 NSCLC patients with EGFR mutation (22%), KRAS mutation (27%) and wild-type profiles (51%), it was observed that 18F-FDG uptake was significantly higher in those harbouring KRAS mutations as compared to EGFR+ or wild-type (SUVmean 9.5 vs. 5.7 vs. 6.6, *p* < 0.001) [17]. These findings are corroborated by a much larger study, encompassing 849 NSCLC patients with 45.9% identified with EGFR mutation, that also showed low SUVmax association with EGFR mutation status [18]. This result could be combined with other clinical factors to improve patient stratification, particularly when EGFR testing is not available [18,19].

A recent study reported on the development of a new PET tracer with high specificity to activating EGFR mutant kinase showing significant correlation between tracer uptake and the EGFR mutation status in both preclinical animal models and in patients with NSCLC [20]. The study aimed to identify, via a new imaging tracer—18F-MPG (*N*-(3-chloro-4-fluorophenyl)-7-(2-(2-(2-(2-^18^F-fluoroethoxy) ethoxy) ethoxy) ethoxy)-6-methoxyquinazolin-4-amine)—, those patients that are sensitive to EGFR-TKIs and to monitor the efficiency of EGFR-TKI therapy. The cut-off value for SUVmax was set at 2.23, showing a greater response to EGFR-TKI in those presenting with SUVmax ≥2.23 as compared to patients with values <2.23 (81.58% vs. 6.06%). Furthermore, 18F-MPG uptake positively correlated with median progression-free survival [20].

While 18F-FDG has its own merits in the functional imaging of lung cancer, it is not the optimal indicator of proliferation, showing poorer correlation with cellular proliferation markers than other PET tracers. Fluoro-3′-deoxythymidine-F18 (18F-FLT), a successfully used imaging marker of cellular proliferation, is a radiolabeled structural analog of a DNA nucleoside—thymidine—and its uptake relates to the activity of thymidine kinase 1 (TK1) that is expressed during DNA synthesis in the S-phase of the cell cycle [21]. The uptake of 18F-FLT in tumour cells is lower as compared to 18F-FDG, as it only accumulates in cells during the S-phase [15]. Yet, several studies demonstrated the superior correlation of 18F-FLT with cellular proliferation markers when compared to the traditional 18F-FDG [22,23]. In one of the first comparative studies that involved a cohort of 26 lung cancer patients, Buck et al. showed high correlation between 18F-FLT uptake and Ki-67 index (*p* < 0.0001; r = 0.92), and concluded that 18F-FLT may be a better imaging marker than FDG for response assessment and outcome prediction [22]. These observations are supported by a recent meta-analysis that assessed 1213 patients from 22 imaging studies that correlated the Ki-67 labeling index with FDG and FLT uptake, respectively, showing that the latter is a more robust marker of tumour proliferation in lung cancer [23]. 

In a recent pilot study, Kairemo et al. demonstrated the feasibility of 18F-FLT PET in monitoring treatment response by early signal activity in NSCLC patients receiving targeted therapies (c-MET inhibitors) [24]. Several others have confirmed the potential of 18F-FLT PET imaging to monitor and guide molecular targeted therapies in NSCLC [25,26,27]. 

Next to the most common Fluor-based radiotracers employed in PET for tumour proliferation imaging, copper is another successful candidate. Functional imaging with PET employing 64Cu-ATSM (Cu-labeled diacetyl-bis(N(4)-methylthiosemicarbazone) and 18F-FDG was undertaken for the intratumoral distribution assessment of the two radionuclides in Lewis lung carcinoma tumour cells implanted in mice [28]. Both proliferation markers (Ki-67 and BrdU-bromodeoxyuridine) and the hypoxic marker, pimonidazole, were used to compare radionuclide uptake with immunohistochemical staining patterns. The association of staining with radionuclide accumulation revealed an increase in Ki-67 positive areas with 18F-FDG uptake increase and, at the same time, a decrease with 64Cu-ATSM accumulation. Conversely, the other proliferation marker, BrdU, showed an opposite behaviour, with the number of BrdU-positive cells being positively correlated with 64Cu-ATSM uptake and negatively related to 18F-FDG accumulation. Given that BrdU is a marker for proliferation by way of DNA synthesis, the fact that cells with high 64Cu-ATSM uptake positively correlated with the number of BrdU cells indicates that they are able to undergo DNA synthesis, though not during the proliferation process (denoted by the low Ki-67 levels which are specific to G1 and early S phase). This result suggests that cells in regions with high 64Cu-ATSM uptake were quiescent, yet sustained DNA synthesis and were sensitive to progression factors, just like quiescent cancer stem cells. Clonogenic assays within the same study have proven the stem-like properties of cells originating from high 64Cu-ATSM uptake tumour areas [28]. Furthermore, pimonidazole-positive areas were specific to regions with low 64Cu-ATSM accumulation, suggestive of mild hypoxic conditions, still optimal for the thriving of clonogenic tumour cells.

This study is a clear illustration of the complexity of the tumour microenvironment and of the many factors that influence tumour development and response to therapy (hypoxia, proliferation, cancer stem cells). Based on the above results, 64Cu-ATSM could potentially serve as a complex imaging biomarker to supply prognostic information for treatment adaptation and optimisation.

#### 2.2.2. Single Photon Emission Computed Tomography (SPECT) Imaging Biomarkers

Beside PET tracers, a number of researchers attempted to develop SPECT radioisotopes for novel insights into EGFR targeting. The capacity of 99mTc-HYNIC-MPG ((2-(2-(2-(2-(4-(3-chloro-4- fluorophenylamino)-6-methoxyquinazolin-7-yloxy)ethoxy)ethoxy)ethoxy)ethyl-6-hydrazinylnicotinate hydrochloride) was evaluated in detecting EGFR-activating mutations both in vitro and in vivo, using human NSCLC cell lines [29]. The study showed that of the four cell lines (EGFR+, EGFR− and wild-type), 99mTc-HYNIC-MPG uptake was the highest in the cell line with exon 19 deletion (PC9), probably due to the activating mutations in EGFR tyrosine kinase domain. The results could serve to further stratify NSCLC patients by identifying the subgroup that would benefit the most from targeted therapies with EGFR-TKIs [29]. 

Table 1 is a compilation of different functional imaging agents tested as markers for tumour proliferation in NSCLC.

#### 2.2.3. Magnetic Resonance Imaging (MRI) Biomarkers

The latest advances in biomaterials, specifically in nanomedicine, have greatly increased the sensitivity of imaging techniques using magnetic resonance to perform accurate and non-invasive functional imaging. In this regard, one of the recent developments is in the field of superparamagnetic iron oxide (SPIO) nanoparticles (40–50 nm), whereby polyethylene glycol-coated SPIO nanoparticles (PEG-SPIO) were synthesized and further labeled with high affinity anti-EGFR monoclonal antibody (cetuximab) for targeted delivery to lung cancer that overexpresses EGFR [30]. The targeting efficiency, MRI contrast enhancement and cytotoxicity of this nanocomposite was evaluated in both H460 lung cancer cells (in vitro) and tumour-bearing rats (H460 lung xenografts) in vivo. The uptake of the nanocomposite in the cell lines was evaluated by Prussian blue staining which showed an increased cellular uptake of anti-EGFR targeted NPs compared to non-targeting NPs at the same iron concentration, suggesting that the high cellular accumulation of anti-EFGR NPs is due to the EGFR receptor-mediated endocytosis pathway. This was also illustrated by TEM (transmission electron microscopy) imaging, where cells incubated with anti-EGFR targeting NPs showed the presence of electron-dense particles in the cell endosome, in contrast with those incubated with non-targeting NPs, which showed no such uptake. To further confirm these results, MRI-based investigation was undertaken by measuring the T2 weighted signal intensity of lung cells after incubation with NPs having various iron concentrations. It was observed that the T2 signal decreased with the increasing iron concentrations in the EGFR targeting NPs group. Furthermore, the signal intensity of lung cancer cells that overexpressed EGFR and were targeted with anti-EGFR-PEG-SPIO decreased more significantly than in the PEG-SPIO (non-targeting NPs) group. The study concluded that efficient identification and targeting of lung cells overexpressing EGFR can be achieved by means of anti-EGFR-PEG-SPIO nanocomposite, under MRI monitoring [30].

### 2.3. Summary of Current Status for Proliferation Biomarkers

While Ki-67 is a marker of proliferation that is well studied in lung cancer, EGFR has a less clear impact and its prognostic role is obscured by new therapies currently employed in clinical practice (such as EGFR-TKIs). To justify further developments in the field of new tracers for EGFR positive NSCLC, also considering the rapid pace of treatment evolution in this subset of patients, a cost–benefit analysis would help clinicians in their decision making. While there are some promising reports, neither the treatment response prediction nor the prognosis of EGFR tumours offered by these biomarkers are convincing enough to support wide clinical implementation.

## 3. Cancer Stem Cells and Imaging Biomarkers

### 3.1. Cancer Stem Cells

Statistics show that recurrence rates among NSCLC remain as high as 30–50%, with low overall 5-year survival rates [31]. One reason for this relatively poor response is the ability of lung cancer cells within the residual disease to regenerate and repopulate the tumour. The power of regeneration is owed to the small fraction of cells with stem-like properties which are phenotypically different from their non-stem counterparts and exhibit vital features for cell survival [32].

Cancer stem cells (CSCs) are a subpopulation of cancer cells that coexist within a tumour with other, non-stem like cells. CSCs have several well-established properties that confer upon them immortality and resistance to both chemo and radiotherapy. Resistance to treatment is multifactorial and is due to the ability of CSCs to efficiently repair DNA damage, to recreate themselves via symmetrical division thus contributing to tumour repopulation, to preferentially reside in specific microenvironmental niches in order to conserve their status, to be recruited into the cell cycle from the quiescent phase, and to exhibit cellular plasticity that enables transformation from CSC to non-CSC state and vice versa [33,34,35].

While the first indicators about the presence of CSCs in lung cancer originate from the early 80s [29], today there are several putative markers for CSCs, from cell surface markers such as CD (cluster of differentiation) molecules, which are surface proteins that enable the analysis of cell differentiation, to aldehyde dehydrogenase (ALDH), an intracellular enzyme and a subset of the CD44+ cells that exhibits high selectivity for CSCs [36]. Overexpression of the hyaluronic acid receptor CD44 was found in neoplasms of epithelial origin, including lung [37,38]. 

Other putative lung CSCs markers that present multipotent characteristics of stem cells are CD166+/CD44+ and CD166+/EpCAM+ (epithelial cell adhesion molecule). Using the above markers, Zakaria et al. showed that isolated lung CSCs exhibit molecular signatures of both normal and cancer stem cells, with biological functions related to angiogenesis, mesenchymal cell differentiation, and cell migration [39].

Another trialed CSC marker in solid tumours is CD133, with several studies demonstrating a link between CD133 expression and stem cell characteristics, including tumour aggressiveness [40,41]. A meta-analysis looking into the prognostic value of the expression of CSC marker CD133 revealed a strong correlation between this marker and prognostic factors among 1004 NSCLC patients [41]. The analysis showed a close correlation of CD133 expression with tumour stage, grade and poor prognosis. On the other hand, Salnikov et al. could not demonstrate any association between CD133 expression and survival of NSCLC patients, despite the indication of CD133 towards a resistant phenotype [42]. Due to such discrepant reports, the prognostic role of CD133 in lung cancer is not fully established, showing the need for the identification of more robust markers.

### 3.2. Imaging Biomarkers for Cancer Stem Cells

Owing to their unique tumour-promoting properties, cancer stem cells must be identified in order to be targeted and eradicated. The identification and targeting of CSCs are greatly dependent on specific markers and/or a combination of markers that are expressed on the surface of cancer stem cells. As CSCs are relatively newly studied descriptors of tumour development and response to therapy, in vivo imaging of CSCs is still in its infancy. Functional imaging studies using CSC-specific radiolabeled markers have been reported for a number of solid cancers, although the majority are reported in tumour-bearing mice [28,43,44,45].

Lewis lung carcinoma tumour cells implanted in mice were evaluated via PET imaging using 64Cu-ATSM and 18F-FDG for the assessment of intratumoral distributions of the radionuclides [28]. Radionuclide uptake was compared with immunohistochemical staining patterns using both proliferation markers (Ki-67 and BrdU) as well as the hypoxic marker, pimonidazole. Furthermore, the clonogenic potential was evaluated via clonogenic assay and compared with 64Cu-ATSM distribution. The study found a direct correlation between tumour regions with high 64Cu-ATSM accumulation and colony forming ability, as cells originating from high 64Cu-ATSM areas had greater colony-forming capacities than those from regions with low and intermediate radionuclide uptake. This shows that 64Cu-ATSM has the potential to serve as a CSC-affinic imaging biomarker, identifying radioresistant tumour areas that could preferentially be targeted with more aggressive agents/techniques.

Another imaging approach tested for CSC identification in NSCLC is MRI, via magnetic nanoparticles. Zhou et al. synthesized a multifunctional peptide–fluorescent–magnetic nanocomposite to be used for in vivo live fluorescence imaging and magnetic resonance imaging in lung tumour xenografts [45]. Owing to their great versatility and applicability, magnetic iron oxide (Fe_3_O_4_) nanoparticles (NPs) are widely studied and used from MRI to cancer therapy. One of the greatest advantages of these magnetic NPs is their flexibility to be designed and synthesized as multifunctional NPs, by adapting the surface ligands according to the intended application [45]. Specific binding peptides for lung cancer stem cells, named as HCBP-1, have been previously identified and validated by the same group, via flow cytometry and fluorescence microscopy [46], being now modified on the surface of fluorescent magnetic nanoparticles to be used for MR imaging of CSCs. The effectiveness of the NPs was tested on cultured human lung cancer cell line (H460) injected in nude mice. Flow cytometry results indicated the potential of NPs to isolate HCBP-1 positive cells in vitro, while in vivo live fluorescent imaging and MRI showed that the multifunctional nanocomposite could serve as an imaging marker for CSC identification [45].

While the number of imaging studies undertaken in lung tumours using biomarkers for CSCs is limited, they open new avenues towards personalised treatment and identify gaps that could promote further research in this field. Among imaging techniques, perhaps the most relevant for further human trials are functional imaging methods employing PET/CT, SPECT and MRI.

The field of functional imaging is continuously growing with new radionuclides (PET) and magnetic nanoparticles (MRI) that have affinity towards CSCs, which could assist in the quantitative assessment of these cancer stem cells within a tumour.

### 3.3. Summary of Current Status for CSC Biomarkers

Most of the current evidence on the value of CSC biomarkers is based on proof-of-concept studies. Since clinically applicable techniques for noninvasive CSC imaging in NSCLC are lacking, taking the existing pre-clinical research of CSC biomarkers to the next level is greatly desirable. To be clinically implementable, there is need for CSC markers with high sensitivity and specificity, which also allow for high-resolution monitoring. With well-designed biocompatible markers, identification and targeting of cancer stem cells using functional imaging techniques could be the next step towards personalised therapy in oncology.

## 4. Circulating Tumour Cells and Imaging Biomarkers

### 4.1. Circulating Tumour Cells and Distant Metastasis

Circulating tumour cells (CTC) are epithelial malignant cells detached from the primary tumour that underwent the epithelial–mesenchymal transition (EMT) and gained the ability to intravasate into the blood stream, migrate to distant anatomic regions and extravasate to favourable metastatic sites. The CTC population is heterogenous and consists of various cellular sub-populations with different phenotypes and functional features, including the capacity of clustering with other blood cells such as leukocytes and platelets. Owing to their ability to convert from one state to the other via EMT, the CTC population includes a subset of multipotent cells with stem-like properties, that were described above as cancer stem cells, and in this context of circulating tumour cells they are the ones responsible for cancer dissemination and formation of micrometastases [47]. Furthermore, CTC clusters, also known as circulating tumour microemboli, were shown to increase the metastatic potential in lung cancer patients [48].

While distant metastasis is known as a final-stage event during cancer progression, experimental studies have shown that cancer cells can actually spread to distant anatomic sites even at early stages of cancer development [49]. Furthermore, there are ways of detecting CTCs from the peripheral blood of patients (so called liquid biopsy) with early stage neoplasms, which might be indicative of tumour aggressiveness and treatment outcome [50]. Given that a number of studies showed a direct correlation between the quantity and types of CTCs detected in blood and patient survival, CTCs could offer an important insight into disease progression and treatment prediction [51].

More research into the role of CTCs reveals important insights into various correlations between factors influencing tumour development and treatment outcome prediction in NSCLC. As discussed above, the identification of EGFR mutations in advanced NSCLC patients is a critical aspect of patient stratification for optimal targeted therapies. While tumour tissue is the commonly preferred standard sample for the evaluation of EGFR mutations, for many patients such samples are not available, which is the reason why a study has been undertaken to search for a surrogate marker for EGFR status through a more accessible way [52]. Circulating-free tumour DNA from plasma/serum samples of NSCLC patients was found to correlate with EGFR mutation with high concordance rate (94.3%) and specificity (99.8%). 

Isolation and detection of CTCs is not without challenges as the capturing technique of these cells from blood must be highly sensitive and specific, which is the reason why different methods of CTC isolation often provided conflicting results. Traditionally, the definition of circulating tumour cells encompasses three components; accordingly, a CTC is a cell that is (1) negative for the hematopoietic cell marker CD45; (2) positive for cytokeratin, a structural protein expressed by epithelial cells; and (3) positive for the epithelial cell adhesion molecule EpCAM, an epithelial cell surface marker [53]. Having these properties as a starting point, a number of techniques have been developed to isolate and quantify the CTC population from blood samples.

CTCs are currently detected in the peripheral blood at a single cell level, with the most commonly employed techniques in lung cancer being the CellSearch^®^ system, the CTC chips or the Isolation by Size of Epithelial Tumour Cells (ISET) filter device. Both the CellSearch^®^ system and the CTC chips employ the EpCAM (epithelial cell specific adhesion molecule) to capture CTCs. The detection rate varies as a function of lung cancer type (NSCLC or SCLC) and stage, with NSCLC patients presenting with lower counts of CTCs than those diagnosed with SCLC, even in late stages of the disease [54]. This observation was explained by the possibly higher fraction of CTCs in NSCLC patients that undergone the EMT, which in turn, led to downregulation of EpCAM expression. In these situations, a CTC detection technique that is independent of EpCAM—such as ISET or the CTChip^®^, which exploits size-based differences between CTCs and hematopoietic cells—could offer more reliable results [55,56].

Studies to date show the potential of circulating tumour markers (such as CTCs and circulating tumour DNA) to serve as surrogates or markers on their own to provide treatment response monitoring, prognosis prediction, detection of early recurrence, etc., which warrants further research in evaluating their role in NSCLC.

### 4.2. Circulating Tumour Cells as Biomarkers in NSCLC

Due to their highly heterogeneous nature, imaging of circulating tumour cells at a single time point might not be relevant for outcome prediction or treatment monitoring, which is the reason why established functional imaging techniques are not adequate for this task. Instead, to evaluate all steps involved in tumour metastasis, continuous monitoring of the primary tumour and of CTCs is recommended [57]. In support of this idea, Wyckoff et al. have transfected both metastatic and non-metastatic rat-derived mammary adenocarcinoma cell lines with green fluorescent protein to quantify tumour cell density in the blood, individual cells in the lung as well as lung metastases. Cells were viewed minute-by-minute using time-laps confocal imaging and revealed the fact that both metastatic and non-metastatic cells display protrusive behaviour; however, metastatic cells showed greater intravasation potential and larger numbers originating from the primary tumour [57]. Over the years, in vivo flow cytometry was developed to increase the time resolution and to create a more dynamic picture of the metastatic process [58].

One of the latest technologies for real-time in vivo imaging of CTCs and CSCs employs multiphoton microscopy and antibody conjugated quantum dots [59]. The study has showed promising results in identifying CTCs with high metastatic potential in mice and concurrently measuring the number, velocity and trajectories of CTCs in the bloodstream. Due to the unique fluorescence signal exhibited by CTCs, this experiment allowed the study of a CTC subpopulation via antibody conjugated quantum dots using various wavelength emissions [59]. To enable direct imaging, tumours (human pancreatic cell line) were grown on the earlobes of mice, thus allowing visualization of blood vessels, of tumour growth over time and CTC detection in the blood vessels near the solid tumour 1 week after inoculation. Metastatic sites were detected in the stomach and intestines. Cancer stem cells in the blood, as a subpopulation of CTCs, were identified through labeling with monoclonal CD24 antibodies conjugated on quantum dots. CSCs were found both in the peripheral tumour tissue as well as on the solid tumour, accumulating specifically on one side of the solid tumour. This observation, whereby CSCs cluster in certain parts of the tumour, suggests the potential of better targeting.

High definition imaging of CTCs in NSCLC was undertaken by means of automated digital microscopy using fluorescent labeling, with the aim of quantifying the CTCs and evaluating their prognostic value in lung cancer patients [60]. For CTC detection, cells were incubated with anti-Cytokeratin antibodies and pre-conjugated anti-CD45 antibody. The detection method offered cytomorphologic evaluation of the cells, looking for Cytokeratin positive and at the same time CD45 negative cells, with a high nuclear cytoplasmic ratio and a large size (compared to other cells in the blood sample). The clinical study encompassed 28 NSCLC patients with evidence of distant metastasis, with all patients receiving chemotherapy or EGFR kinase inhibitor. For CTC evaluation, blood specimens were collected periodically (overall, 66 specimens at various time periods), over 12 months. CTCs were detected in 68% of samples and no differences in prevalence or quantity was found between adenocarcinomas and squamous cell carcinomas. During the time course of the study, an increase in CTC prevalence was observed, from 56% of specimens presenting CTCs in the first month of enrollment, to 63% after 3 months and a further increase to 94% at 6 months and afterwards. A cut-off value of 5 CTCs/mL was chosen to correlate the CTC count with outcome (survival). As such, patients with ≥ 5 CTCs/mL had a median survival of 244 days, while in those patients with < 5 CTCs/mL the median survival was not reached at a median follow-up of 304 days. Patients with high CTC counts had a hazard ratio for death of 4.0, relative to those with low counts (*p* = 0.0084) [60]. CTCs could, therefore, serve as potential biomarkers for patient stratification and risk assessment, contingent on the availability of high precision CTC detection and quantification assays. 

Based on the premise that both CTC counts and metabolic parameters defined by 18F-FDG can be correlated with patient prognosis, a number of studies combined the two techniques (PET imaging and CTCs quantification) to find possible relationships between them. A multi-center study that included 71 NSCLC patients (all stages, predominantly early-stage) who underwent 18F-FDG PET imaging was designed to evaluate CTCs from samples within 90 days and prior to surgery or radio-chemotherapy [61]. CTCs were quantified by a non-EpCAM based method, using immunofluorescence (cytokeratins, CD45, DAPI-staining for nuclear quantitation). The results revealed that while FDG uptake via SUVmax was strongly dependent on tumour stage and histology, no such association was found for CTCs, suggesting that the two biomarkers may act in a complementary manner. Furthermore, the identification of many individual and clustered CTCs in early-stage disease (characterized by weak FDG uptake) may not be indicative of distant metastasis formation. The association between tumour glucose metabolism and CTCs could be influenced by the heterogeneity of CTCs and might depend on the CTC subpopulation type [61].

A prospective biomarker trial that enrolled 53 patients with advanced NSCLC found no correlation between circulating tumour DNA (cell-free DNA) and metabolic tumour volume or total lesion glycolysis based on FDG PET imaging, hypothesizing that cell-free DNA may be representative of more complex biological mechanisms [62]. In a similar study, Morbelli et al. evaluated the correlation between circulating tumour DNA counts and PET parameters, both locoregionally and at distant sites, showing a positive correlation between cell-free DNA base line levels and tumour metabolic activity [63]. As only SUVmax was associated with circulating DNA, the authors concluded that this biomarker may be more reflective of tumour metabolism and biologic behaviour than tumour burden in advanced NSCLC. 

The association of CTCs with early relapse in resected NSCLC was analysed using PET images from 102 patients both before and 1 month after radical resection [64]. CTCs were detected in 39.2% of patients before surgery and in 27.5% after the resection, which was strongly correlated with SUVmax and pathological stage. The presence of CSCs post-surgery was also associated with a shorter recurrence free survival, irrespective of staging.

A recent study reported preliminary data on a cohort of 17 metastatic NSCLC patients that underwent 18F-FDG PET imaging with the aim of finding a correlation between CTC numbers (determined with the ISET method) and clinical/metabolic parameters. The results indicated a strong association of CTCs present in blood with tumour uptake characterized by SUVmean [65]. CTCs were detected in 59% of patients with a mean of 3 CTCs/mL (1–7 range), with a lower number of CTCs found in patients that underwent chemotherapy. 

Based on the study findings, which are corroborated by data from previous reports, it was suggested that the combination between CTC quantification and FDG PET parameters could offer an improved prognostic stratification of NSCLC patients [65].

While to date the number of studies is limited, identification and quantification of CTCs in NSCLC could have an important impact on the evaluation of treatment response and overall prognosis. Studies have indicated that treatment of NSCLC can influence the CTC population both negatively and positively [66]. Mobilization of CTCs after radiotherapy, surgery or systemic treatment might either lead to cell eradication and improved tumour control, or may promote metastasis, in which case CTCs should be targeted and eliminated. Martin et al. showed that in patients with advanced NSCLC treated with palliative intent using large doses of radiation, CTC numbers increased after treatment. Many of these cells presented with high levels of DNA damage, as identified by γH2AX assay, suggesting that the damaged CTCs originated from the irradiated tumour [67]. CTCs isolated from post-irradiation blood samples showed viability through in vitro proliferation. This observation justifies the need for further studies into therapy-triggered CTC mobilization and the development of efficient systemic therapies to specifically target CTCs to overcome the formation of micrometastases.

### 4.3. Summary of Current Status for CTC Biomarkers

The role of CTCs as prognostic biomarkers is already well understood. Future developments in CTC detection in clinical practice will occur together with liquid biopsy studies of circulating tumour DNA (ctDNA). While CTCs correlation with PET parameters often show conflicting results (or weak correlations) among existing studies, further investigations are required in order to understand patient heterogeneity among NSCLC sufferers. Moreover, identification and biological characterization of CTCs could offer real-time monitoring of personalised targeted therapies in combination with functional imaging modalities.

## 5. Imaging Biomarkers for Apoptosis

Apoptosis, or programmed cell death, is a key physiological feature that ensures tissue homeostasis in normal conditions. Evasion of apoptosis is one of the hallmarks of cancer. It is acknowledged that numerous effects of radio- and chemotherapy are mediated by apoptosis, including resistance to treatment through altered apoptosis, upregulation of anti-apoptotic signals and downregulation of pro-apoptotic ones [68]. For instance, decrease in p53 signalling is an indicator of apoptosis evasion. As a tumour suppressor protein, p53 regulates cell cycle and has the potential to induce apoptosis as a response to various cellular signalling. Mutations in p53 signalling pathways lead to uncontrolled proliferation and inhibition of apoptosis. Similarly, proteins of the Bcl-2 family are important regulators of programmed cell death. Borner et al. examined the expression of the p53 and Bcl-2 family proteins in 49 specimens of patients with NSCLC via immunostaining, showing a negative influence of Bcl-2 expression on relapse-free survival (*p* = 0.02), while the expression of p53 and Bcl-2 was significantly associated with metastasis-free survival (*p* < 0.01) [69]. The authors concluded that Bcl-2 family proteins have no clear or direct impact on clinical outcome owing to their complex interaction with the apoptotic pathway.

The insulin-like growth factor 1 receptor (IGF-1R) is a transmembrane receptor tyrosine kinase overexpressed in neoplasms, having an anti-apoptotic effect through enhancement of survival and proliferation [70]. Furthermore, IGF-1R expression was shown to be activated in cancers that are resistant to EGFR inhibitors, including lung cancer [71]. High expressions of IGF-1R were associated with poor disease-free survival in NSCLC [72]. Being identified as a potential diagnostic and therapeutic biomarker, IGF-1R is currently assessed from a non-invasive imaging perspective. When labelled with 111In for SPECT imaging, IGF-1 showed good selectivity for tumour cells and strong correlation with IGF-1R expression in human breast cancer cells, suggesting potential application in the molecular imaging of other carcinomas [73]. 

Imaging of apoptotic pathway can serve for treatment response monitoring after radio/chemotherapy through the evaluation of apoptotic death rate. Imaging biomarkers developed for programmed cell death include annexin V labelled with common PET radionuclides such as 11C, 18F, 64Cu and 68Ga [74,75]. Most of these radio-compounds are in pre-clinical evaluation. Owing to activation of caspase-3 during apoptotic death, radiolabelled caspase-3 was tested as a substitute for annexin V. The first human study designed for apoptotic imaging involved eight subjects and employed an 18F-labelled PET tracer (18F-ML-10), demonstrating efficient binding to apoptotic sites, and favourable biodistribution as well as safety profile [76].

### Summary of Current Status for Apoptosis Biomarkers

The role of apoptosis in cancer development and response to therapy is well established. Although apoptosis is acknowledged as a promising target for anticancer therapy, imaging biomarkers of apoptosis are still in their early days of development, as most radiolabelled markers have not seen clinical applications. As far as lung cancer is concerned, even pre-clinical studies on apoptotic cell death imaging are scarce, requiring translation from other anatomical sites that showed promising results. 

## 6. Conclusions

There is no doubt that, nowadays, the field of oncology is strongly oriented towards personalised treatment, irrespective of the type of cancer. The latest insights into the biological and radiobiological properties of tumours and their cellular sub-populations have offered the possibility to develop and clinically implement specific tracers and markers, allowing for more accurate diagnosis, treatment planning and delivery [77]. NSCLC patients are also gaining from these advances, starting from the discovery of EGFR mutations which confer sensitivity to tyrosine kinase inhibitors. The refinement of lung cancer subtypes and their corresponding therapies have further improved patient outcome. 

The world of new radiobiological tracers is greatly stimulating, owing to the possibility of studying the heterogeneity of lung cancers, of stratifying tumours by their prognostic characteristics and of overcoming the limit of biopsy that often does not allow a complete and exhaustive description of the biology of such tumours. The clinical management of tumour heterogeneity is a significant challenge as tumour response is dictated by the particular behaviour of each sub-group of cancer cells. Cellular heterogeneity given by proliferation kinetics, stemness, hypoxia or other factors calls for specific markers and targeting; therefore, the near future of biomarkers will rely on complementarity rather than a common solution valid for all (radio)biological particularities of a tumour [78].

This paper focused on three main factors influencing tumour kinetics (development and proliferation of primary tumour) and tumour dynamics (infiltration and distant metastases) in the context of NSCLC: tumour proliferation, cancer stem cells and circulating tumour cells. While advances in knowledge cover all these aspects, there is potential for improvement on the clinical side to better understand tumour resistance to chemotherapy, to augment the efficiency of immunotherapy for primary as well as metastatic cancers, and to design clinical trials that employ specific biomarkers to identify and tackle resistant tumour sub-populations. The near future will likely bring further developments in the emerging areas, such as cancer stem cell biomarkers, where research is still in pre-clinical stages, whereas in the more established fields—of proliferation and tumour progression—research has advanced into clinical phases, with the expectation of more refined utilization and wider implementation.

## Figures and Tables

**Table 1:** Functional imaging biomarkers for tumour proliferation in non-small cell lung cancer (NSCLC).

Study Aim [Ref]	Study Type	Proliferation Marker/ Targeting Agent	Comments
**Positron Emission Tomography**			
Proliferation imaging with ^18^F-FLT vs. ^18^F-FDG [Buck et al. (2003)] [22]	Prospective study (26 patients with pulmonary nodules)	**Proliferation marker**: Ki-67 **Targeting agent**: ^18^F-FLT ^18^F-FDG	A highly significant correlation (*p* < 0.0001) and a high correlation coefficient (r = 0.92) was observed between ^18^F-FLT uptake and Ki-67 index, while the correlation coefficient between Ki-67 and ^18^F-FDG was weak (r = 0.59). No FLT uptake was detected in non-proliferating tumours.
PET imaging for EGFR mutation evaluation and response to treatment [Sun et al. (2018)] [20]	Preclinical rodent model; Clinical NSCLC study	**Proliferation marker**: EGFR **Targeting agent**: ^18^F-MPG	A greater response to EGFR-TKI was found in patients with SUV_max_ ≥ 2.23 (81.58% vs. 6.06%). Median progression-free survival was also longer (348 days) in the cohort with SUV_max_ ≥ 2.23 than in SUV_max_ < 2.23 (183 days). ^18^F-MPG PET for quantification of EGFR-activating mutation status could identify patients sensitive to EGFR-TKIs.
Evaluation of the role of ^64^Cu-ATSM in PET imaging [Oh et al. (2009)] [28]	In vivo mice study (Lewis lung carcinoma tumour cells implanted in mice)	**Proliferation markers**: Ki-67 BrdU **Targeting agent**: ^64^Cu-ATSM ^18^F-FDG	Tumour regions with high ^18^F-FDG but low ^64^Cu-ATSM uptake correlated with increase in Ki-67. On the other hand, the number of BrdU-positive cells were positively correlated with ^64^Cu-ATSM uptake and negatively related to ^18^F-FDG accumulation. This suggests that cells in regions with high ^64^Cu-ATSM uptake were quiescent, yet were sensitive to progression factors, like quiescent CSCs.
**Single Photon Emission Computed Tomography**			
Evaluation of ^99m^Tc-HYNIC-MPG for detection of EGFR-activating mutations [Xiao et al. (2017)] [29]	In vitro cell line study (human NSCLC cell lines EGFR+/- and wild-type); In vivo animal xenograft model	**Proliferation marker**: EGFR **Targeting agent**: ^99m^Tc-HYNIC-MPG	^99m^Tc-HYNIC-MPG uptake was the highest in the cell line with exon 19 deletion (PC9), probably due to the activating mutations in EGFR tyrosine kinase domain. SPECT imaging with ^99m^Tc-HYNIC-MPG could potentially identify NSCLC patients that would benefit the most from targeted therapies with EGFR-TKIs.
**Magnetic Resonance Imaging**			
EGFR targeting with active iron oxide NP for MRI [Wang et al. (2017)] [30]	H460 lung cancer cells (in vitro) and tumour-bearing rats (H460 lung xenografts) in vivo.	**Proliferation marker**: EGFR **Targeting agent**: Anti-EGFR-polyethylene glycol-superparamagnetic iron oxide (anti-EGFR-PEG-SPIO)	Both in vitro and in vivo MRI studies showed the potential of anti-EGFR-labeled iron oxide nanoparticles to identify and target lung cells that overexpress EGFR. The study had both imaging and therapeutic (theranostic) goals achieved with anti-EGFR targeting based on magnetic nanoparticles using MRI and focused ultrasound ablation.

**Abbreviations:** EGFR = epidermal growth factor receptor; PET = positron emission tomography; CSCs = cancer stem cells; MRI = magnetic resonance imaging; NPs = nanoparticles.
